# Supplementary material for: Macrophages foster anti-tumor immunity by ZEB1-dependent cytotoxic T cell chemoattraction
Source: Commun Biol. 2025 Jul 1;8:976. doi: 10.1038/s42003-025-08339-7 (PMC12218307; doi:10.1038/s42003-025-08339-7)
Supplement: Supplementary file 2 — Supplementary Information [file 42003_2025_8339_MOESM2_ESM.pdf]

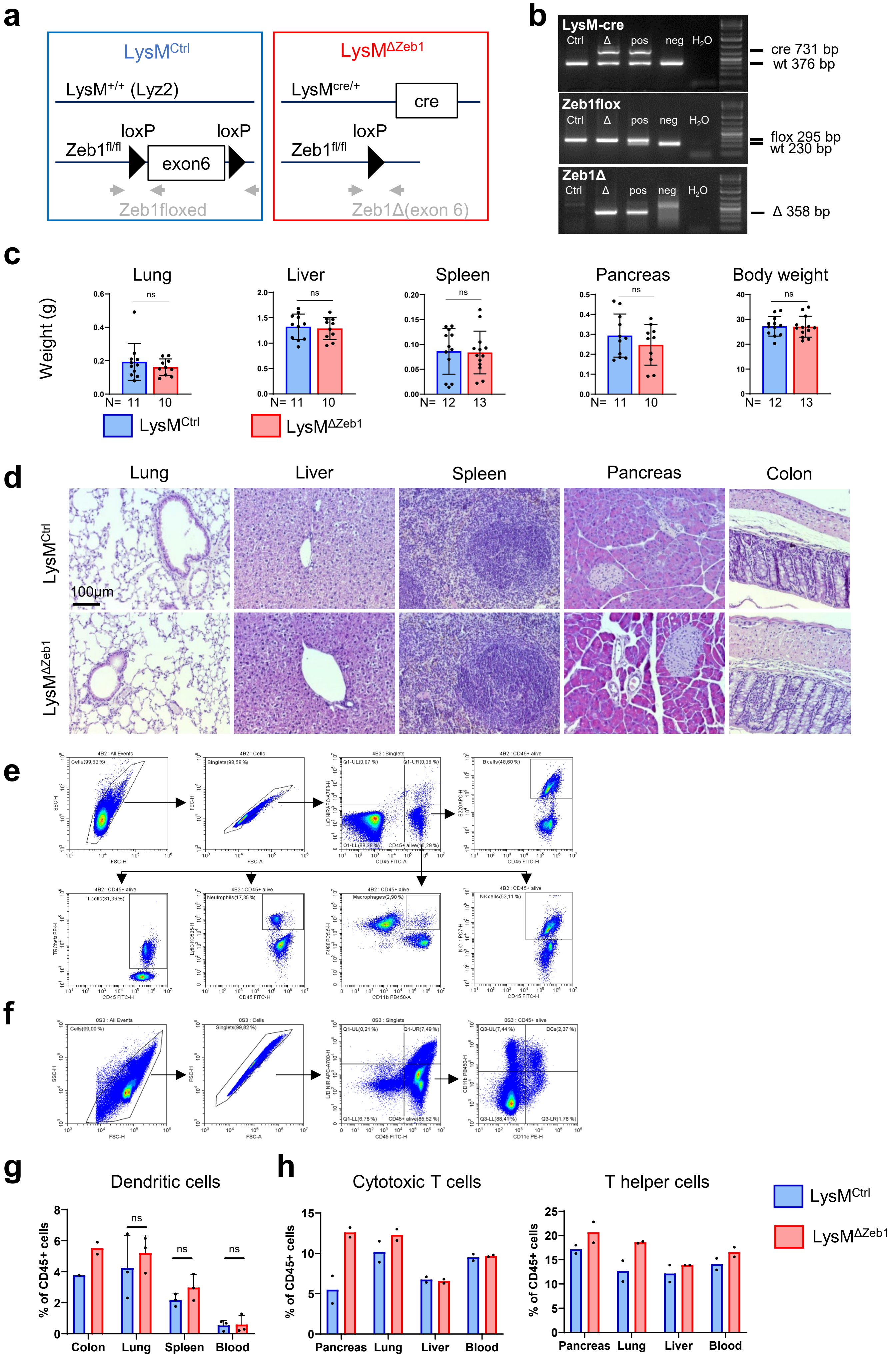

a
LysMCtrl
LysM+/+ (Lyz2)
loxP
loxP
Zeb1fl/fl
exon6
Zeb1floxed
LysMΔZeb1
LysMcre/+
cre
loxP
Zeb1fl/fl
Zeb1Δ(exon 6)
b
LysM-cre
Ctrl Δ pos neg H2O
cre 731 bp
wt 376 bp
Zeb1flox
Ctrl Δ pos neg H2O
flox 295 bp
wt 230 bp
Zeb1Δ
Ctrl Δ pos neg H2O
Δ 358 bp
c
Weight (g)
Lung
Liver
Spleen
Pancreas
Body weight
ns
N= 11 10
N= 11 10
N= 12 13
N= 11 10
N= 12 13
LysMCtrl
LysMΔZeb1
d
Lung
Liver
Spleen
Pancreas
Colon
LysMCtrl
LysMΔZeb1
100µm
e
f
g
Dendritic cells
% of CD45+ cells
Colon Lung Spleen Blood
h
Cytotoxic T cells
% of CD45+ cells
Pancreas Lung Liver Blood
T helper cells
% of CD45+ cells
Pancreas Lung Liver Blood
LysMCtrl
LysMΔZeb1

Figure S1: Targeting of *Zeb1* in LysM-expressing cells does not cause major phenotypic abnormalities

a. Schematic of the *Zeb1* targeting strategy in LysM-positive cells. Grey arrows mark primer annealing sites for genotyping. **b**. PCR genotyping of DNA isolated from $LysM^{Ctrl}$ (Ctrl) and $LysM^{\Delta Zeb1}$ (Δ) mice. wt = wild type, Δ= deleted, pos = positive control, neg = negative Ctrl, bp = base pairs. **c**. Weight of organs and animals of $LysM^{Ctrl}$ and $LysM^{\Delta Zeb1}$ mice (n indicated in figure; means +SD; t-test). **d**. Representative images of H&E stained $LysM^{Ctrl}$ and $LysM^{\Delta Zeb1}$ organs. **e**. Gating strategy for flow cytometry of immune cell subtypes in organs and blood of $LysM^{Ctrl}$ and $LysM^{\Delta Zeb1}$ mice (referring to Fig. 2a). **f**. Gating strategy for flow cytometry of dendritic cells in organs and blood of $LysM^{Ctrl}$ and $LysM^{\Delta Zeb1}$ mice (referring to Fig. S1g). **g-h.** Percentage of immune cells of $LysM^{Ctrl}$ and $LysM^{\Delta Zeb1}$ organs, as determined by flow cytometry (n($LysM^{Ctrl}$ / $LysM^{\Delta Zeb1}$)= 1/ 2 colon; n=3 other organs (**g**); n=2 (**h**); mean +SD, where applicable; two-tailed t-test). ns: not significant.

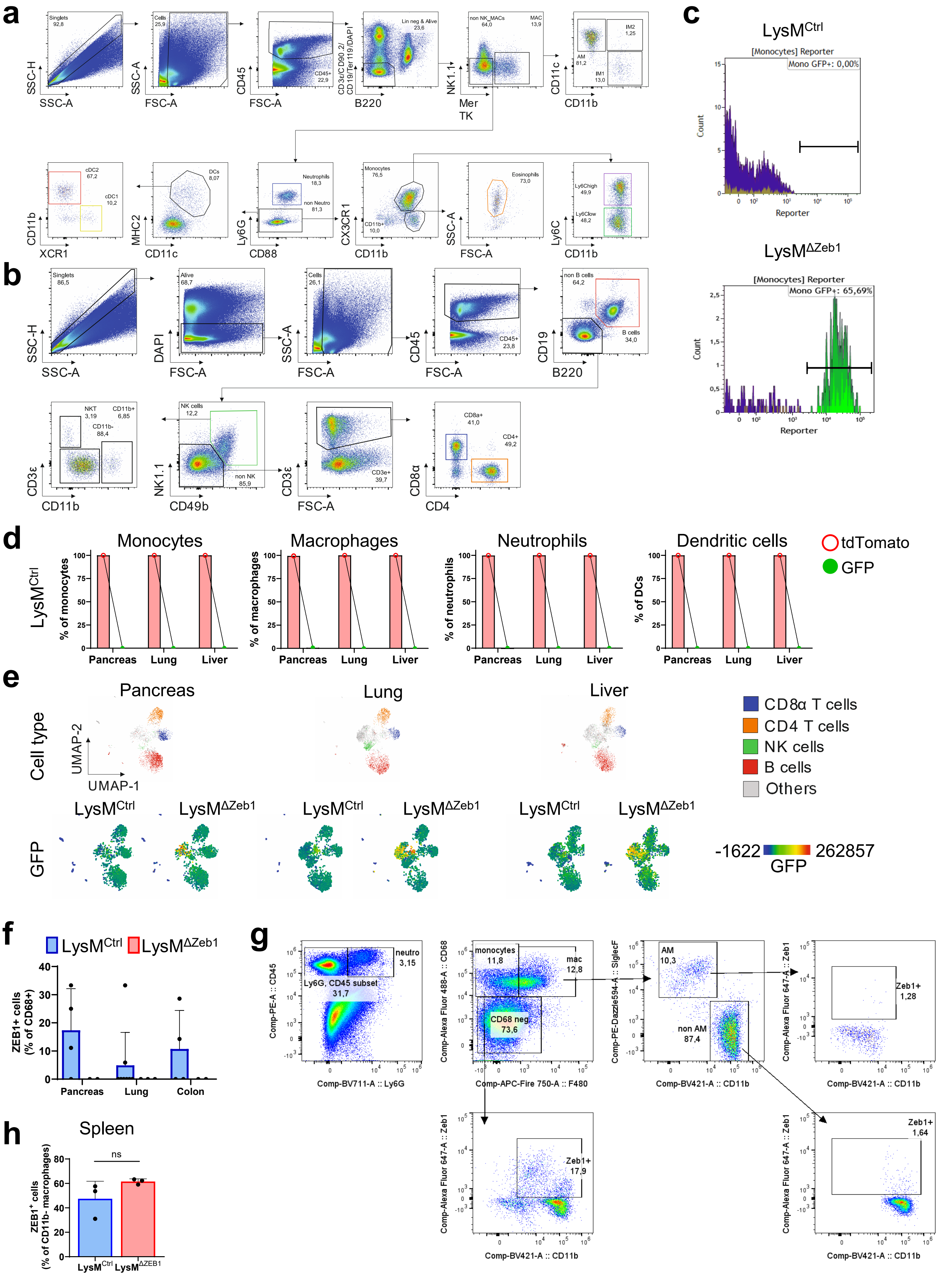
a
b
c
d
e
f
g
h
LysMCtrl
LysMΔZeb1
[Monocytes] Reporter
Mono GFP+: 0,00%
Mono GFP+: 65,69%
Monocytes
Macrophages
Neutrophils
Dendritic cells
tdTomato
GFP
Pancreas
Lung
Liver
Colon
Cell type
UMAP-1
UMAP-2
CD8α T cells
CD4 T cells
NK cells
B cells
Others
-1622
262857
ZEB1+ cells (% of CD68+)
Spleen
ns
ZEB1+ cells (% of CD11b- macrophages)

Figure S2: Validation of LysM-Cre activity and ZEB1 loss in macrophages

a-b. Gating strategy for flow cytometry of myeloid (**a**; referring to Fig. 2b, c, S2d) and lymphoid (**b**; referring to Fig. S1h and S2e) cells isolated from mT/mG+ $LysM^{Ctrl}$ and $LysM^{\Delta Zeb1}$ mice. **c**. Representative histogram of GFP intensities in flow cytometry of cells isolated from mT/mG+ $LysM^{Ctrl}$ and $LysM^{\Delta Zeb1}$ mice used for Fig. 2c and S2d after gating according to Fig. S2a or S2b. **d**. Percentage of tdTomato+ or GFP+ cells in immune cell subtypes of mT/mG-positive $LysM^{\Delta Zeb1}$ (n=2). **e**. Flow cytometry UMAP clustered cells isolated from organs of mT/mG+ $LysM^{Ctrl}$ and $LysM^{\Delta Zeb1}$ mice. GFP expression is depicted as color gradient (n=2 each). **f**. Scoring of CD68+;ZEB1+cells from IF stained tissue from mT/mG-negative $LysM^{Ctrl}$ and $LysM^{\Delta Zeb1}$ mice from Fig. 2e. **g**. Gating strategy for flow cytometry of intracellular ZEB1 in alveolar (AM) and non-AM (non-AM) macrophages in lungs of $LysM^{Ctrl}$ and $LysM^{\Delta Zeb1}$ mice (referring to Fig. 2f). **h**. Fraction of CD45+;Ly6C-;CD11b-;ZEB1+ macrophages in spleens of $LysM^{Ctrl}$ and $LysM^{\Delta Zeb1}$ mice, as determined by flow cytometry (referring to Fig. 2f; mean ±SD; n=3; two-tailed t-test; ns: not significant).

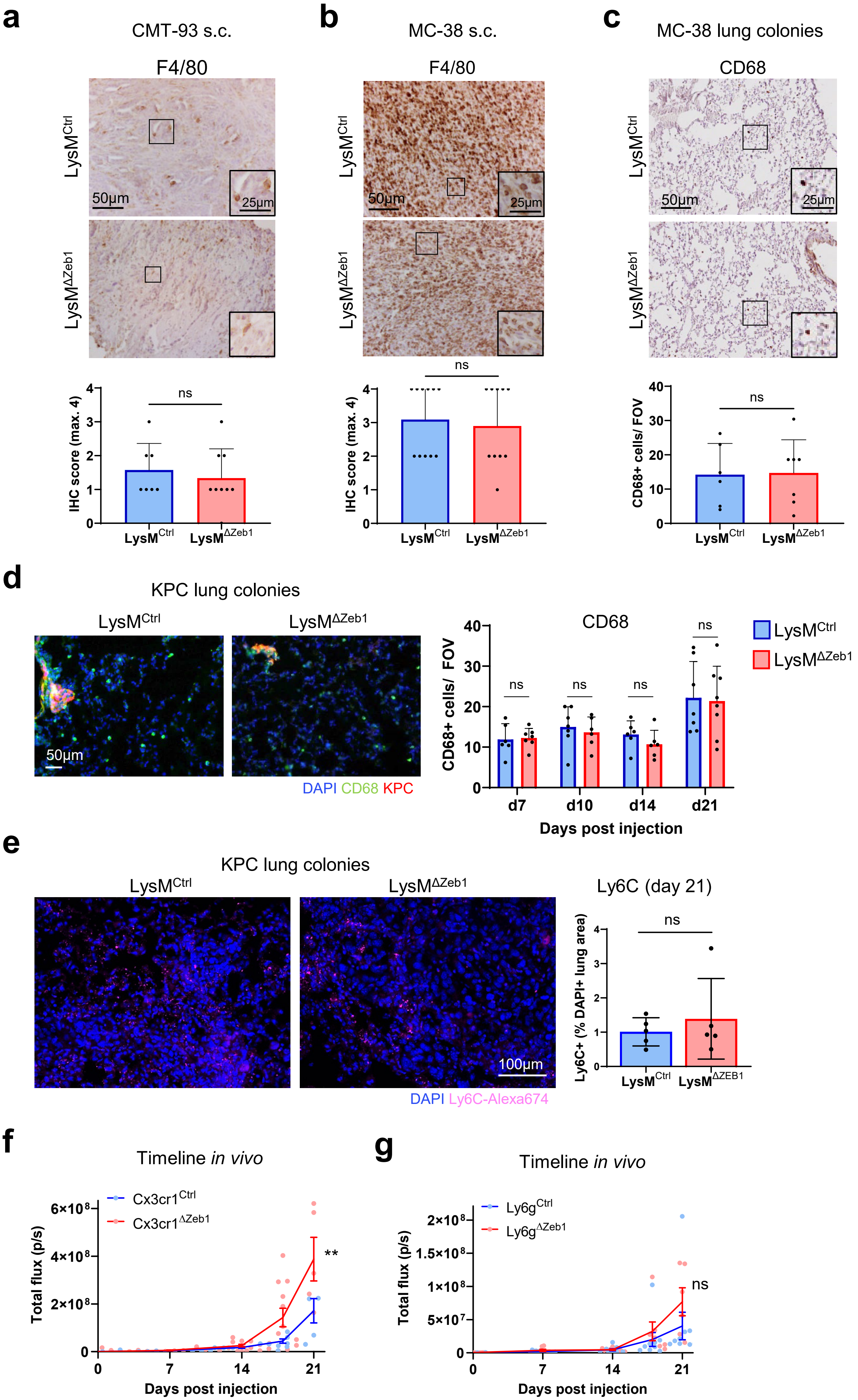

a
CMT-93 s.c.
F4/80
LysMCtrl
LysMΔZeb1
50µm
25µm
IHC score (max. 4)
ns
b
MC-38 s.c.
F4/80
c
MC-38 lung colonies
CD68
CD68+ cells/ FOV
d
KPC lung colonies
DAPI CD68 KPC
Days post injection
e
Ly6C (day 21)
DAPI Ly6C-Alexa674
100µm
Ly6C+ (% DAPI+ lung area)
LysMΔZEB1
f
Timeline in vivo
Cx3cr1Ctrl
Cx3cr1ΔZeb1
Total flux (p/s)
**
g
Ly6gCtrl
Ly6gΔZeb1

Figure S3: Loss of ZEB1 does not affect intratumoral macrophage/monocyte infiltration in LysM-Cre mice and moderately enhances lung colonization in *Zeb1*$^{\mathit{flox/flox}}$;*Cx3cr1-Cre*$^{Cre/+}$ and *Zeb1*$^{\mathit{flox/flox}}$;*Ly6g*$^{\mathit{Cre\text{-}Tom/+}}$ mice

a-d. Representative images and quantifications of IHC or IF stainings for F4/80 and CD68 of s.c. CMT-93 (**a**), s.c. MC-38 (**b**) tumors and MC-38 (**c**) and KPC (**d**) lung colonies in LysM^{Ctrl} and LysM$^{\Delta Zeb1}$ mice. Insets show higher magnification. **e**. Representative images and quantifications of IF staining of Ly6C in KPC lung colonies (means ±SD). **f-g**. *In vivo* BLI signal of tail vein injected KPC tumor cells over time in Cx3cr1^{Ctrl} (n=6) and Cx3cr1$^{\Delta Zeb1}$ (n=12) mice (**f**) or Ly-6g^{Ctrl} (n= 9) and Ly-6g$^{\Delta Zeb1}$ (n=7) mice (**g**); means ±SEM ns: not significant; **:p<0.01; two-tailed t-test (a, b, c, e); 2-way ANOVA (d, f, g).

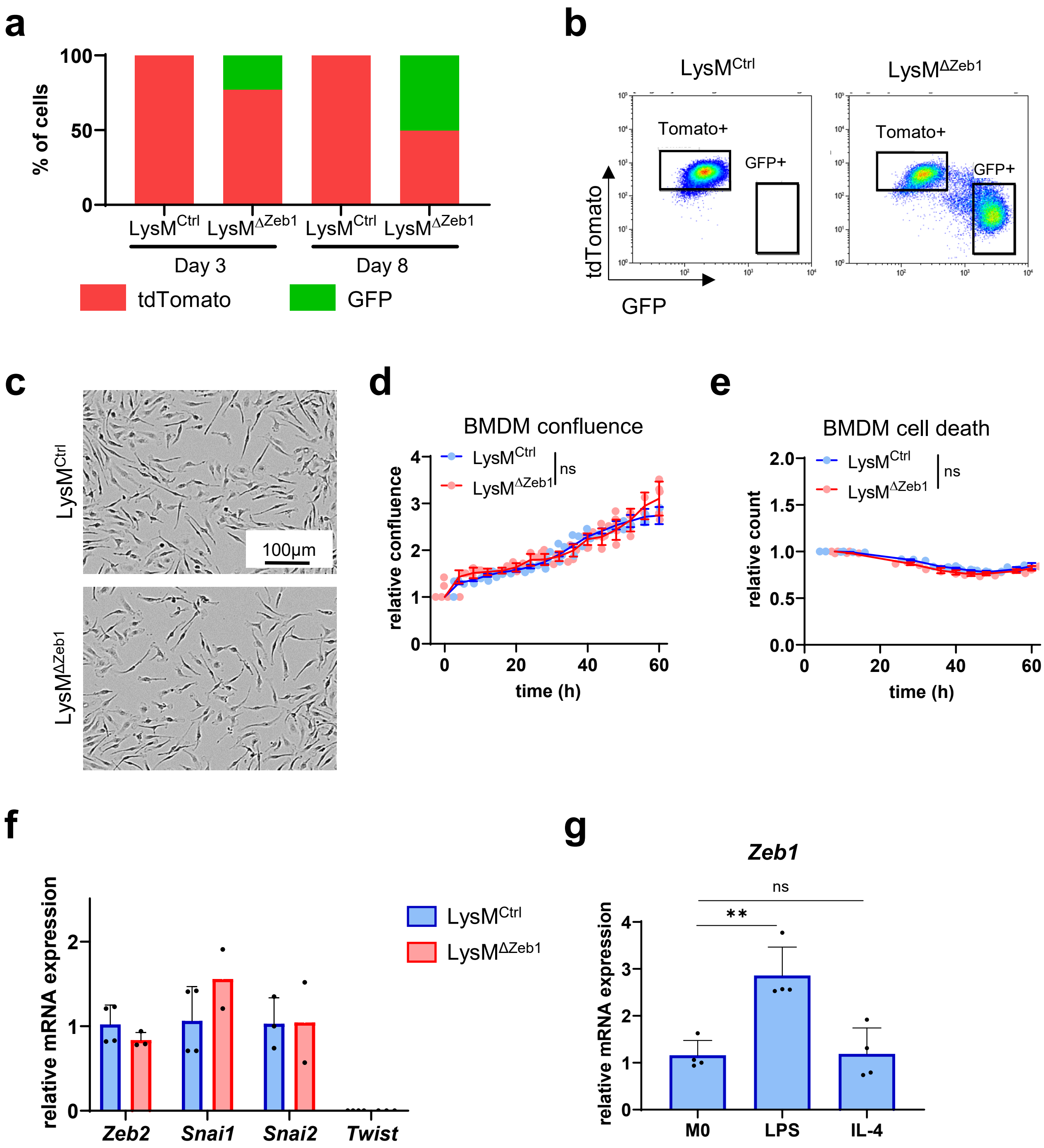
a
% of cells
100
50
0
LysMCtrl LysMΔZeb1 LysMCtrl LysMΔZeb1
Day 3
Day 8
tdTomato
GFP
b
LysMCtrl
LysMΔZeb1
Tomato+
GFP+
tdTomato
GFP
c
LysMCtrl
LysMΔZeb1
100µm
d
BMDM confluence
relative confluence
time (h)
ns
e
BMDM cell death
relative count
time (h)
ns
f
relative mRNA expression
Zeb2
Snai1
Snai2
Twist
g
Zeb1
ns
**
relative mRNA expression
M0
LPS
IL-4

Figure S4: ZEB1 is dispensable for BMDM cultivation and upregulated upon LPS stimulation

a. Flow cytometry of $LysM^{Ctrl}$ and $LysM^{\Delta Zeb1}$ BMDMs with mT/mG reporter at 3 and 8 div ($n>1.15*10^5$ cells). **b**. Representative gating for sorting $LysM^{Ctrl}$ and $LysM^{\Delta Zeb1}$ BMDMs using the mT/mG reporter. **c**. Representative bright field images of cultured $LysM^{Ctrl}$ and $LysM^{\Delta Zeb1}$ BMDMs. **d**. Relative confluence of $LysM^{Ctrl}$ and $LysM^{\Delta Zeb1}$ BMDMs over time (n=3). **e**. Relative cell death of $LysM^{Ctrl}$ and $LysM^{\Delta Zeb1}$ BMDMs as determined by SYTOX uptake (n=3). **f**. Relative expression levels of indicated mRNAs of sorted $LysM^{Ctrl}$ and $LysM^{\Delta Zeb1}$ BMDMs (n≥2). **g**. Relative Zeb1 mRNA expression levels of sorted $LysM^{Ctrl}$ and $LysM^{\Delta Zeb1}$ BMDMs after LPS or IL-4 stimulation (n=4). Mean ±SD; **:p<0.01; 2-way ANOVA (**d**, **e**); two-tailed t-test (**f**), 1-way ANOVA (**g**).

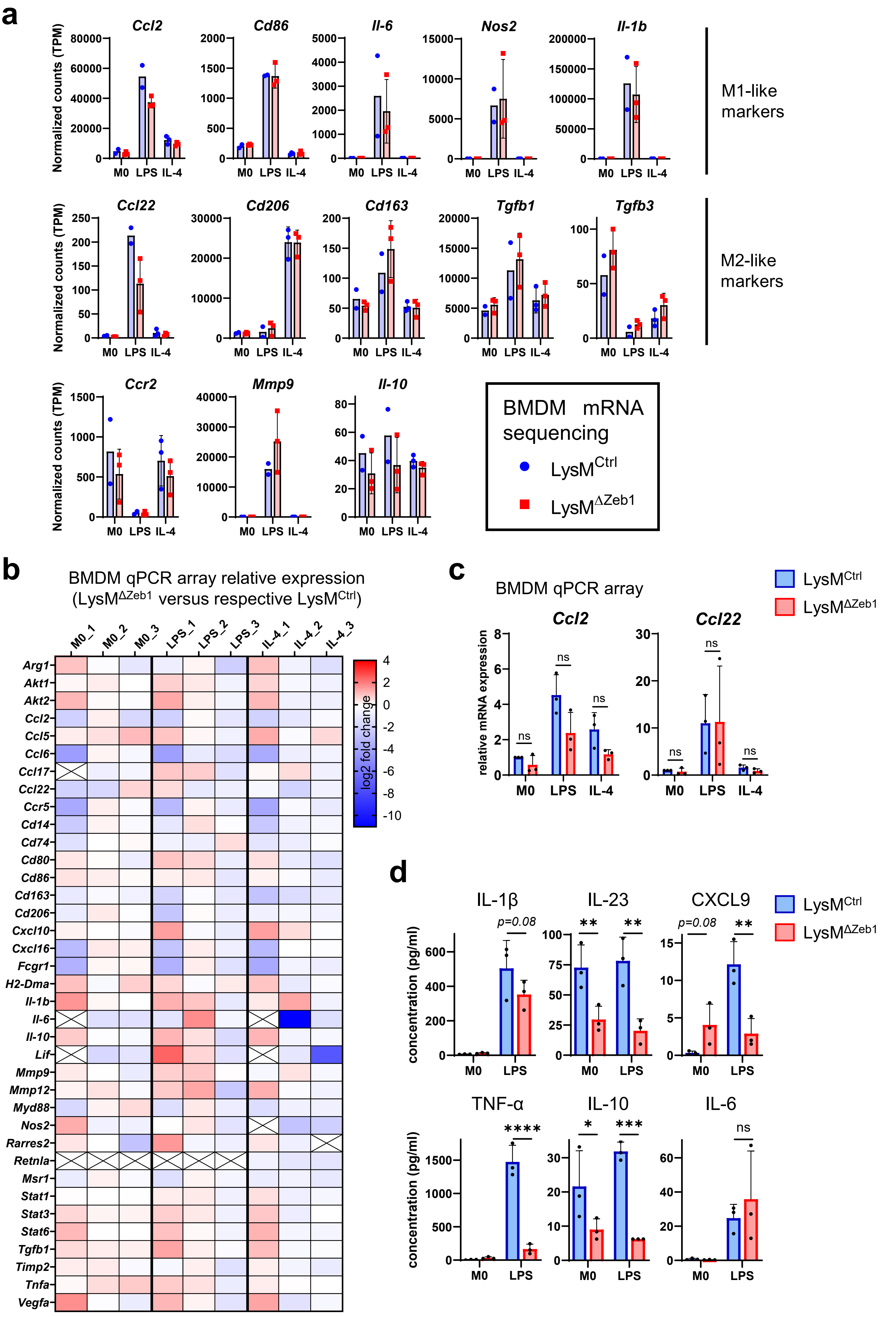
a
Ccl2
Cd86
Il-6
Nos2
Il-1b
M1-like markers
Ccl22
Cd206
Cd163
Tgfb1
Tgfb3
M2-like markers
Ccr2
Mmp9
Il-10
Normalized counts (TPM)
M0 LPS IL-4
BMDM mRNA sequencing
LysM^Ctrl
LysM^ΔZeb1
b
BMDM qPCR array relative expression
(LysM^ΔZeb1 versus respective LysM^Ctrl)
M0_1 M0_2 M0_3 LPS_1 LPS_2 LPS_3 IL-4_1 IL-4_2 IL-4_3
Arg1
Akt1
Akt2
Ccl2
Ccl5
Ccl6
Ccl17
Ccl22
Ccr5
Cd14
Cd74
Cd80
Cd86
Cd163
Cd206
Cxcl10
Cxcl16
Fcgr1
H2-Dma
Il-1b
Il-6
Il-10
Lif
Mmp9
Mmp12
Myd88
Nos2
Rarres2
Retnla
Msr1
Stat1
Stat3
Stat6
Tgfb1
Timp2
Tnfa
Vegfa
log2 fold change
4 2 0 -2 -4 -6 -8 -10
c
BMDM qPCR array
Ccl2
Ccl22
relative mRNA expression
ns
M0 LPS IL-4
LysM^Ctrl
LysM^ΔZeb1
d
IL-1β
IL-23
CXCL9
TNF-α
IL-10
IL-6
concentration (pg/ml)
p=0.08
**
****
*
***
ns
M0 LPS
LysM^Ctrl
LysM^ΔZeb1

Figure S5: Expression of selected polarization markers and cytokines in $LysM^{Ctrl}$ and $LysM^{\Delta Zeb1}$ BMDMs

a. Normalized counts as transcripts per million (TPM) of indicated selected genes in $LysM^{Ctrl}$ and $LysM^{\Delta Zeb1}$ BMDMs either unstimulated (n=2/3 for $LysM^{Ctrl}$/$LysM^{\Delta Zeb1}$) or stimulated with LPS (n=2/3 for $LysM^{Ctrl}$/$LysM^{\Delta Zeb1}$) or IL-4 (n=3/3 for $LysM^{Ctrl}$/$LysM^{\Delta Zeb1}$), as derived from bulk RNA sequencing (means ±SD). **b**. Differentially expressed genes in unstimulated or LPS or IL-4 stimulated $LysM^{\Delta Zeb1}$ compared to $LysM^{Ctrl}$ BMDMs as measured by a customized qPCR array and depicted in log_2 fold change of expression. X marks non-detectable mRNA levels. All transcripts were normalized to Gapdh (n=3). **c**. Relative mRNA expression of Ccl2 and Ccl22 in $LysM^{Ctrl}$ and $LysM^{\Delta Zeb1}$ BMDMs (n=3; means ±SD; 2-way ANOVA). **d**. Quantification of indicated intracellular cytokine levels in pg/ml per 10 µg of protein lysates obtained from untreated (M0) or LPS-treated $LysM^{Ctrl}$ and $LysM^{\Delta Zeb1}$ BMDM using a bead-based immunoassay (n=3; means ±SD; 2-way ANOVA; ns: not significant; *:p<0.05; **:p<0.01; ***:p<0.001; ****:p<0.0001).

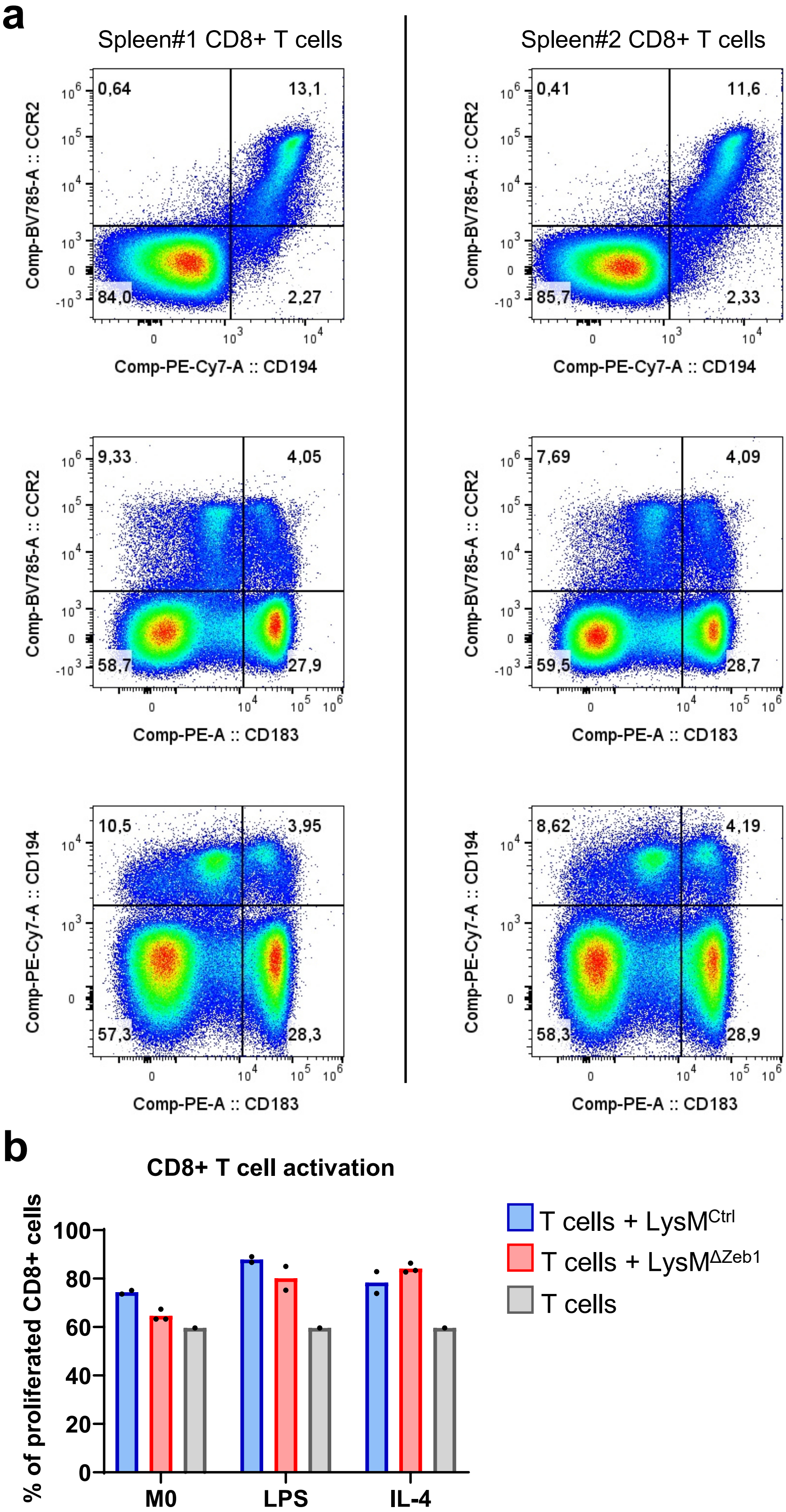
a
Spleen#1 CD8+ T cells
Spleen#2 CD8+ T cells
Spleen#3 CD8+ T cells
Comp-BV785-A :: CCR2
Comp-PE-Cy7-A :: CD194
Comp-PE-A :: CD183
0,64
13,1
84,0
2,27
0,41
11,6
85,7
2,33
0,78
24,0
73,1
2,19
9,33
4,05
58,7
27,9
7,69
4,09
59,5
28,7
19,3
5,02
56,3
19,4
10,5
3,95
57,3
28,3
8,62
4,19
58,3
28,9
20,1
4,90
55,1
19,8
b
CD8+ T cell activation
% of proliferated CD8+ cells
M0
LPS
IL-4
T cells + LysMCtrl
T cells + LysMΔZeb1
T cells

Figure S6: ZEB1 in macrophages has no major effect on proliferation of naive splenic CD8+ T cells which show coupled expression of CCR2 (CD192) and CCR4 (CD194) but not CXCR3 (CD183)

a. Flow cytometry of splenic CD8+ T cells from $LysM^{Ctrl}$ mice (n=3) isolated via magnetic bead separation after surface staining of CD192 (CCR2), CD914 (CCR4) and CD183 (CXCR3). Percentages of the gated cells among all viable CD8+ T cells are indicated. **b**. Percentage of proliferated (*i.e.*, activated) CD8+ T cells as among total CD8+ T cells in absence (n=1) or presence of M0, LPS or IL-4 pre-stimulated $LysM^{Ctrl}$ (n=2) or $LysM^{\Delta Zeb1}$ (n=3) BMDMs (means; 2-way ANOVA).

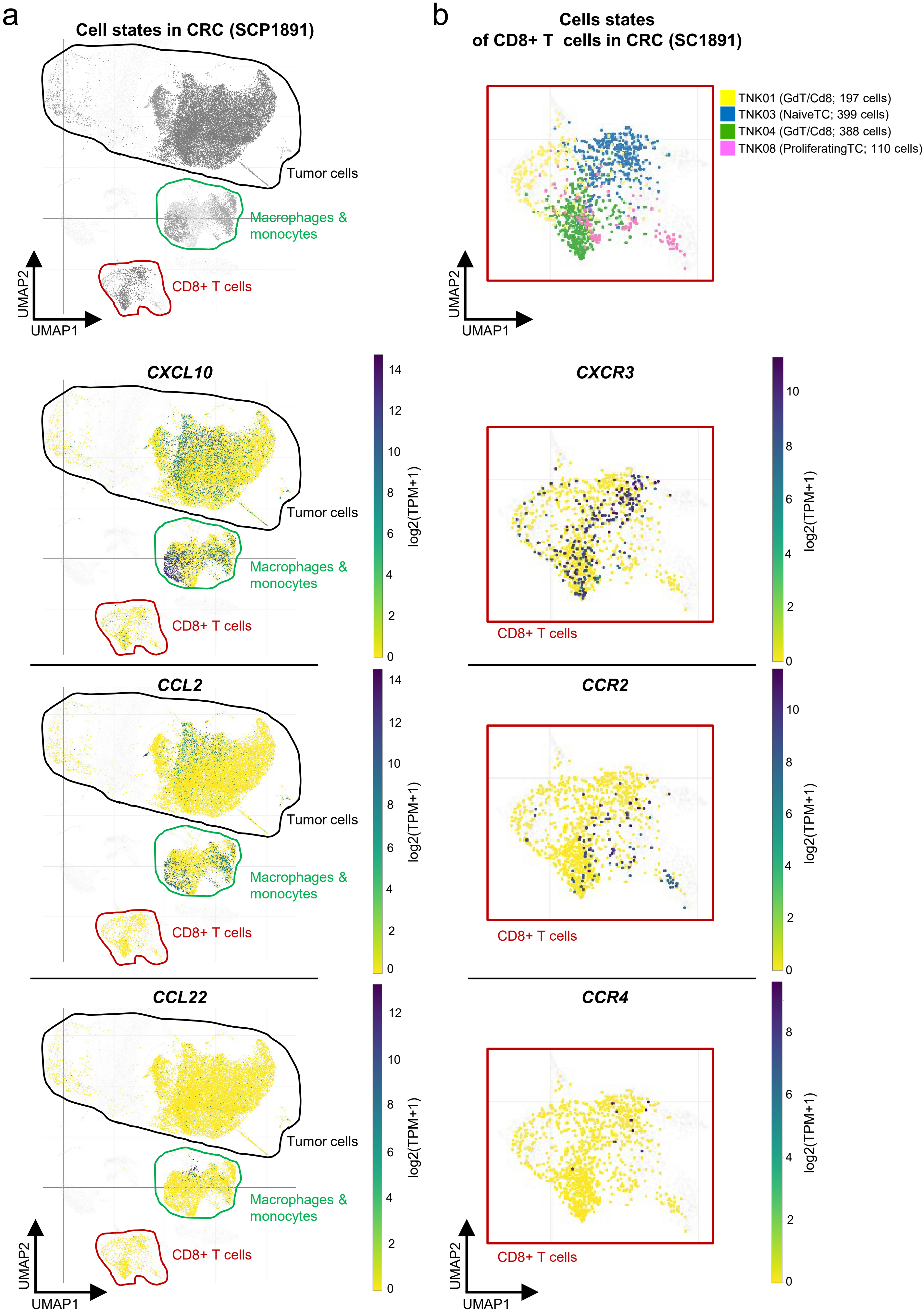

a
Cell states in CRC (SCP1891)
Tumor cells
Macrophages & monocytes
CD8+ T cells
UMAP2
UMAP1
b
Cells states of CD8+ T cells in CRC (SC1891)
TNK01 (GdT/Cd8; 197 cells)
TNK03 (NaiveTC; 399 cells)
TNK04 (GdT/Cd8; 388 cells)
TNK08 (ProliferatingTC; 110 cells)
CXCL10
CCL2
CCL22
CXCR3
CCR2
CCR4
log2(TPM+1)

Figure S7: *CXCL10*, *CCL2* and *CCL22* are expressed in subsets of macrophages/monocytes and *CXCR3*, *CCR2* and *CCR4* are expressed in subsets of tumor-infiltrating *CD8*+ T cells in colorectal cancer

a. UMAP plot visualization of cell states (top) and of the expression of the indicated genes in a human CRC single cell RNA sequencing dataset. Plots and annotations were retrieved from the Broad Institute's Single Cell portal (SCP) using the indicated dataset. **b**. UMAP plot visualizations of the indicated genes as in (**a**) specifically in *CD8*+ T cells, as retrieved using SCP's built-in cell type filtering tool.

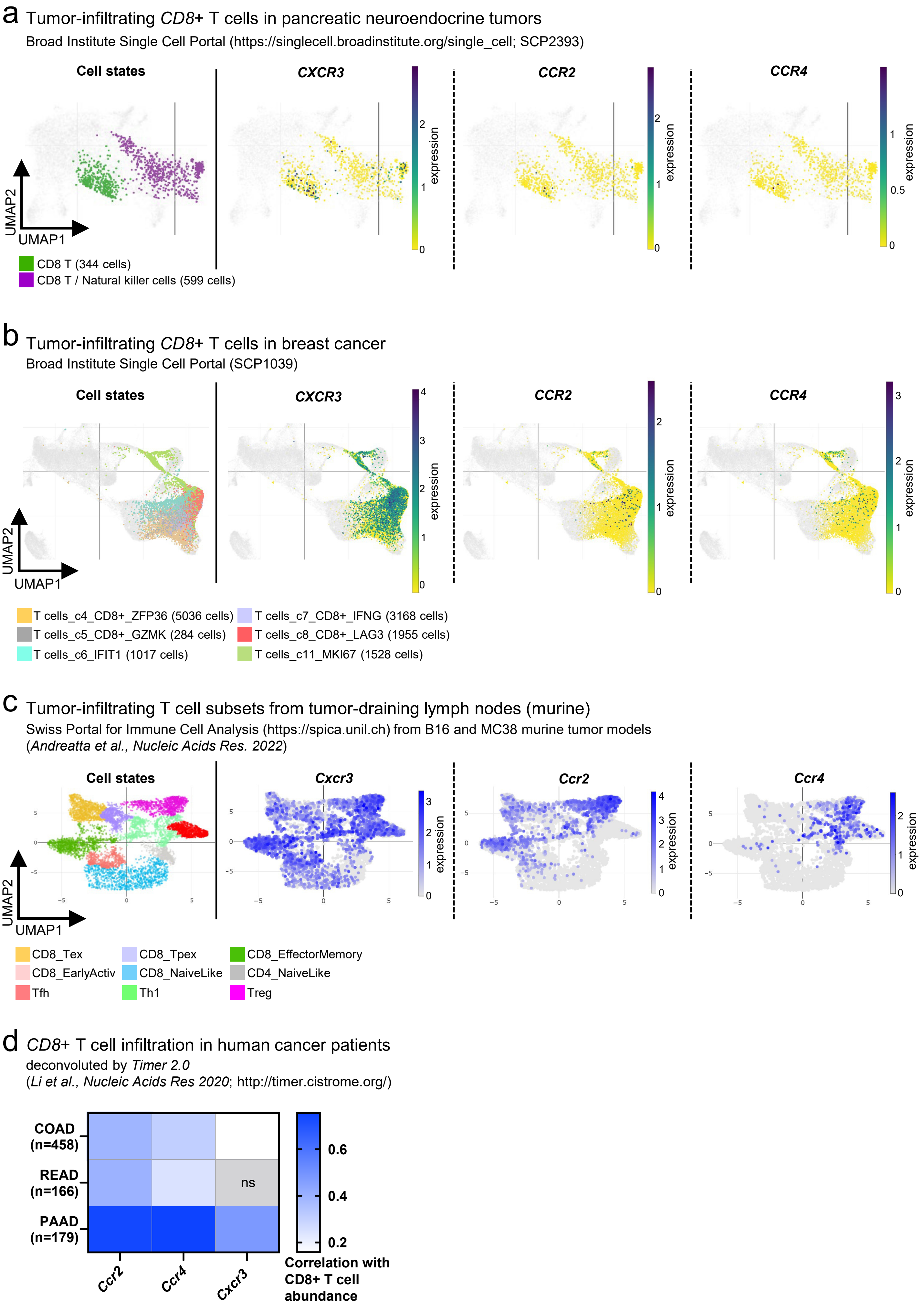
a
Tumor-infiltrating CD8+ T cells in pancreatic neuroendocrine tumors
Broad Institute Single Cell Portal (https://singlecell.broadinstitute.org/single_cell; SCP2393)
Cell states
CXCR3
CCR2
CCR4
expression
UMAP2
UMAP1
CD8 T (344 cells)
CD8 T / Natural killer cells (599 cells)
b
Tumor-infiltrating CD8+ T cells in breast cancer
Broad Institute Single Cell Portal (SCP1039)
Cell states
CXCR3
CCR2
CCR4
expression
UMAP2
UMAP1
T cells_c4_CD8+_ZFP36 (5036 cells)
T cells_c5_CD8+_GZMK (284 cells)
T cells_c6_IFIT1 (1017 cells)
T cells_c7_CD8+_IFNG (3168 cells)
T cells_c8_CD8+_LAG3 (1955 cells)
T cells_c11_MKI67 (1528 cells)
c
Tumor-infiltrating T cell subsets from tumor-draining lymph nodes (murine)
Swiss Portal for Immune Cell Analysis (https://spica.unil.ch) from B16 and MC38 murine tumor models
(Andreatta et al., Nucleic Acids Res. 2022)
Cell states
Cxcr3
Ccr2
Ccr4
expression
UMAP2
UMAP1
CD8_Tex
CD8_EarlyActiv
Tfh
CD8_Tpex
CD8_NaiveLike
Th1
CD8_EffectorMemory
CD4_NaiveLike
Treg
d
CD8+ T cell infiltration in human cancer patients
deconvoluted by Timer 2.0
(Li et al., Nucleic Acids Res 2020; http://timer.cistrome.org/)
COAD (n=458)
READ (n=166)
PAAD (n=179)
ns
Ccr2
Ccr4
Cxcr3
0.6
0.4
0.2
Correlation with CD8+ T cell abundance

Figure S8: *CXCR3*, *CCR2* and *CCR4* are expressed in subsets of tumor-infiltrating *CD8+* T cells in pancreatic and breast cancers

a-b. UMAP plot visualizations of *CD8+* T cell states (left) and of the expression of the indicated genes (right) specifically in these *CD8+* T cell subsets derived from a human pancreatic (**a**) and breast cancer (**b**) single cell RNA sequencing datasets. Plots and annotations were retrieved from the Broad Institute's Single Cell Portal (SCP) using the indicated datasets and SCP's built-in cell type filtering tool. **c.** UMAP plot visualizations of murine T cell states (left) and of the expression of the indicated genes derived from a single cell RNA sequencing dataset generated from T cells in tumor-draining lymph nodes of mice bearing subcutaneous MC-38 and B6 tumors [101]. Note the expression of *Cxcr3*, *Ccr2* and *Ccr4* in subsets of *Cd8+* T cells. Plots and annotations were retrieved from the 'Swiss Portal for Immune Cell Analysis' (https://spica.unil.ch) [59]. **d**. Correlation of CD8+ T cell infiltration in human colon (COAD) rectal (READ) and pancreatic (PAAD) cancer patients with the expression of the indicated genes, as deconvoluted from bulk RNA sequencing using *Timer2.0* (http://timer.cistrome.org/) [60].

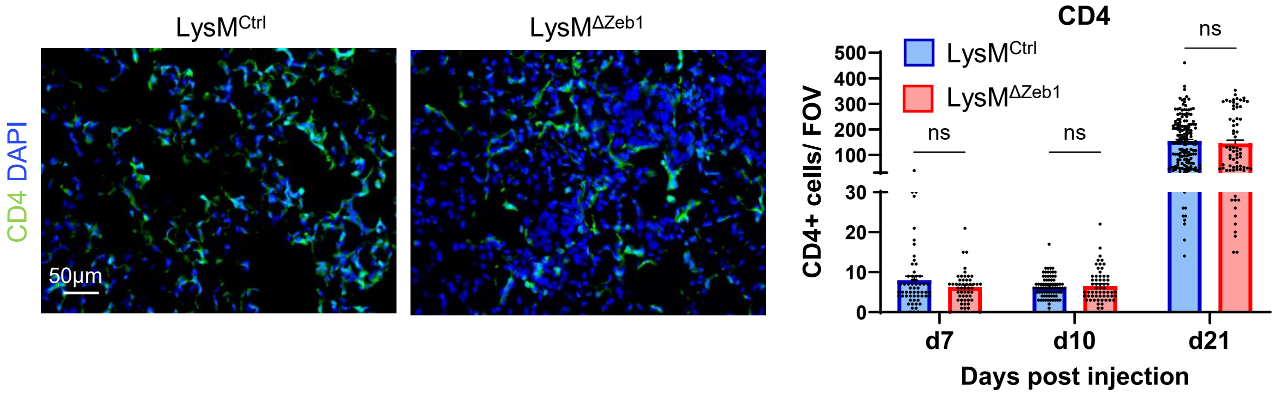

Figure S9: Infiltration of CD4+ cells into lung colonies is unaffected by loss of ZEB1 in macrophages

Representative images at 10 dpi and quantification over time of IF for CD4+ cells of KPC lung colonies in LysM^{Ctrl} and LysM$^{\Delta Zeb1}$ mice with DAPI-stained nuclei (n>50 images; means +SEM; 2-way ANOVA).

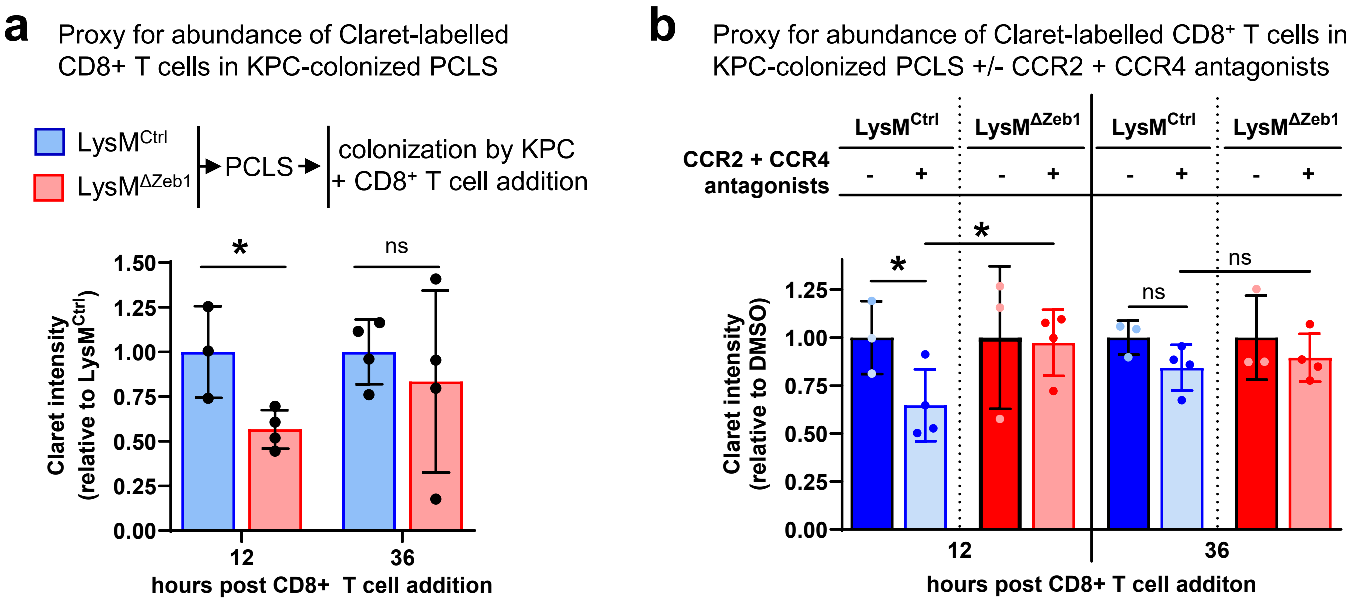

Figure S10: CD8+ T cell abundance is transiently higher in KPC-colonized LysM^{Ctrl} precision-cut lung slices (PCLS) after CD8+ T cell addition (TCA) than in LysM$^{\Delta Zeb1}$ PCLS and is sensitive to CCR2/CCR4 antagonists.

a-b. (referring to Fig. 2b, c, S2d). Measurement of Claret total fluorescence (per lung area) as proxy for CD8+ T cell abundance after CD8+ TCA in KPC-colonized LysM^{Ctrl} and LysM$^{\Delta Zeb1}$ PCLS (**a**) as well as the combination of CCR2 and CCR4 antagonists or DMSO as vehicle control (**b**). Means ±SD; n≥3. *:$p<0.05$; ns: not significant; Two-tailed Welsh t-test (**a**); 2-way ANOVA (**b**).

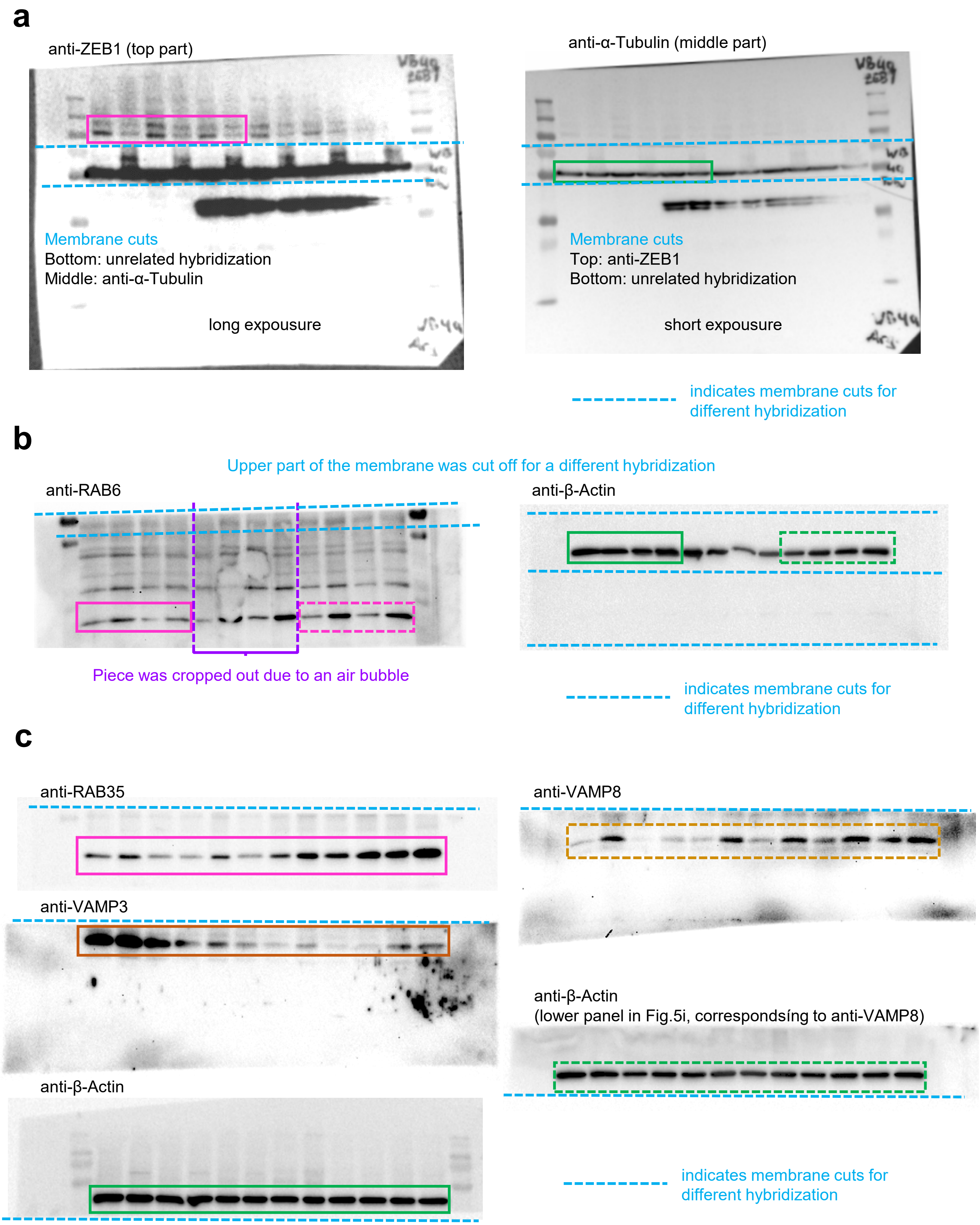
a
anti-ZEB1 (top part)
Membrane cuts
Bottom: unrelated hybridization
Middle: anti-α-Tubulin
long expousure
anti-α-Tubulin (middle part)
Membrane cuts
Top: anti-ZEB1
Bottom: unrelated hybridization
short expousure
indicates membrane cuts for different hybridization
b
Upper part of the membrane was cut off for a different hybridization
anti-RAB6
anti-β-Actin
Piece was cropped out due to an air bubble
indicates membrane cuts for different hybridization
c
anti-RAB35
anti-VAMP8
anti-VAMP3
anti-β-Actin
(lower panel in Fig.5i, correspondsíng to anti-VAMP8)
anti-β-Actin
indicates membrane cuts for different hybridization

d

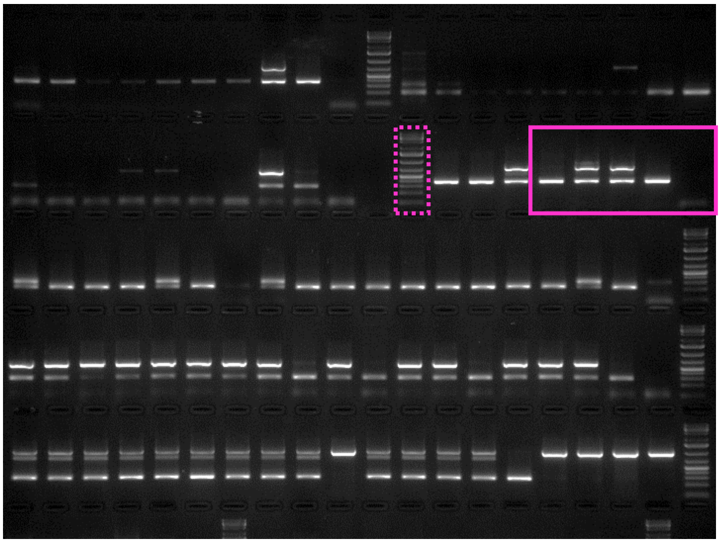

Gel1: LysM-cre

Please note that the DNA ladder (dashed line box) has been moved to the right for the Fig. S1b

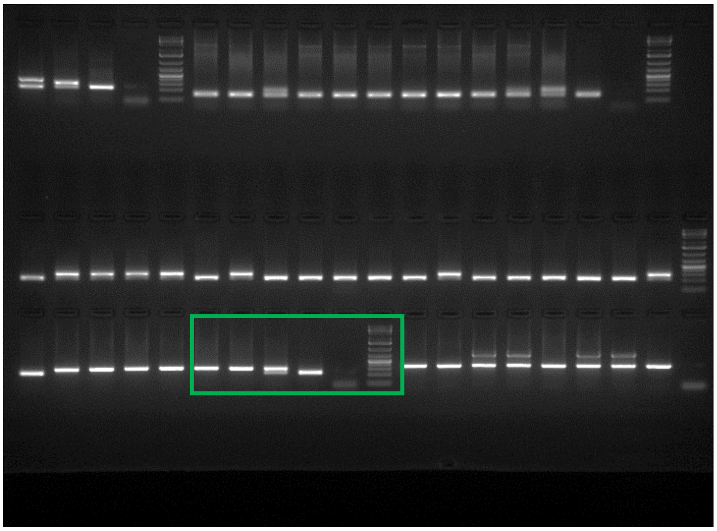

Gel2: Zeb1flox

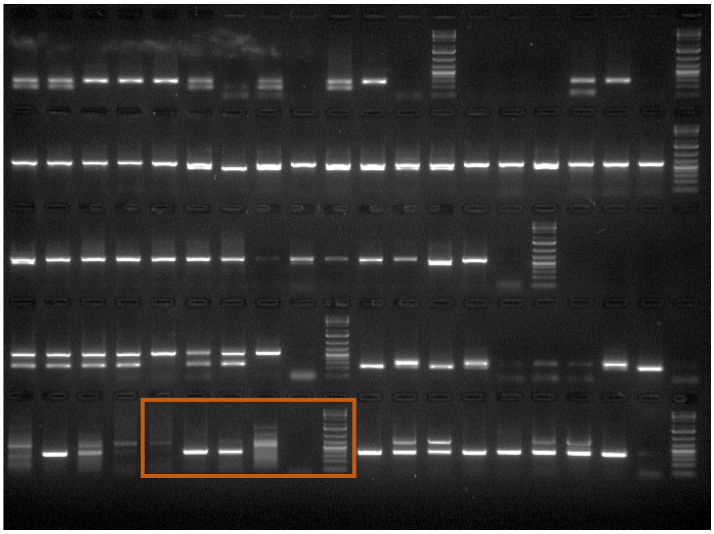

Gel3: Zeb1Δ

Figure S11: Unedited blot and gel images

a-c. Uncropped scans of western bots in Fig. 4c (**a**), 5h (**b**) and 5i (**c**) **d**. Uncropped agarose gel pictures in Fig. S1b.

Supplementary Table 1

Oligonucleotides

Primers for polymerase chain reaction (PCR)

Target	Forward primer	Reverse primer
LysM-Cre	cttgggctgccagaatttctc	agcgattagctggagccatcaag<br>cccagaaatgccagattacg
Zeb1 del	cgtgatggagccagaatctgacccc	gccatctcaccagcccttactgtgc
Zeb1 flox	cgtgatggagccagaatctgacccc	gccctgtctttctcagcagtgtgg<br>gccatctcaccagcccttactgtgc

Primers and UPL numbers for quantitative reverse transcriptase PCR (qRT-PCR)

Target	Forward primer	Reverse primer	UPL #
Gapdh	agcttgtcatcaacgggaag	tttgatgttagtggggtctcg	9
Snai1	cttgtgtctgcacgacctgt	caggagaatggcttctcacc	71
Snai2	cattgccttgtgtctgcaag	agaaaggcttttccccagtg	71
Twist	agctacgccttctccgtct	tccttctctggaaacaatgaca	58
Zeb1	aggtgatccagccaaacg	ggtggcgtggagtcagag	93
Zeb2	ccagaggaaacaaggatttcag	aggcctgacatgtagtcttgtg	42

Antibodies

Primary antibodies

Specificity	Host	Catalog no.	Manufacturer	Dilution
α-Tubulin	mouse	T6199	Sigma-Aldrich	1:5000
β-Actin	mouse	A5441	Sigma-Aldrich	1:5000
CD8	rabbit	50389-T26	Sino Biologicals	1:500
MCP1/CCL2	rabbit	PA115555	Thermo Fisher Sci.	1:600
CD68 (human)	mouse	ab201973	Abcam	1:100
CD68	rabbit	PA5-78996	Invitrogen	1:600
cleaved Caspase-3	rabbit	9664S	Cell Signaling	1:200
F4/80	rat	MCA4976	Bio-Rad	1:200
Ki67	rabbit	ab16667	abcam	1:300
RAB6	rabbit	9625T	Cell Signaling	1:1000

RAB35	rabbit	88244T	Cell Signaling	1:1000
VAMP3	rabbit	13640S	Cell Signaling	1:1000
VAMP8	rabbit	13060S	Cell Signaling	1:1000
ZEB1	rabbit	HPA027524	Sigma-Aldrich	1:1000 (IHC)<br>1:2000 (WB)
ZEB1	rabbit	NBP1-05987	Novus Biologicals	1:250
ZEB1	rabbit	E2G6Y #70512	Cell Signaling	1:400 (IF)
ZEB1	mouse	AMAb90510	Sigma-Aldrich	1:300

Secondary antibodies

Specificity	Host	Catalog no.	Manufacturer	Dilution
AlexaFluor488 anti-rabbit IgG (H+L)	goat	A11034	Sigma-Aldrich	1:200
anti-rabbit-HRP polymer	goat	K4003	DAKO	1:1
anti-rat-HRP	rabbit	A18915	life technologies	1:500
CF640R anti-rabbit IgG (H+L)	goat	SAB4600164	Sigma-Aldrich	1:200
CF640R anti-mouse IgG (H+L)	goat	SAB4600343	Sigma-Aldrich	1:200
anti-rabbit-HRP (WB)	goat	111-035-144	Dianova	1:10000
anti-mouse-HRP (WB)	goat	115-035-146	Dianova	1:10000

FACS antibodies

Specificity	Catalog no.	Manufacturer	Dilution
TruStain FcX™ PLUS (anti-mouse CD16/32)	156603	BioLegend	1:200
Purified anti-mouse CD16.2 (9E9)	149502	BioLegend	1:400
InVivoMAb anti-mouse CD16/CD32 (2.4G2)	BE0307	Bio X Cell	1:400
B220-APC	103211	BioLegend	1:200
CCR3-APC	144511	BioLegend	1:200
CD3e-Biotin	100304	BioLegend	1:200
CD3e-Al647	100322	BioLegend	1:200
CD4-Al488	100532	BioLegend	1:50
CD4-APC-Fire750	100568	BioLegend	1:400
CD4-BV605	100547	BioLegend	1:200
CD8a-Al647	100727	BioLegend	1:200
CD8a-BV570	100740	BioLegend	1:400
CD11b-Al700	101222	BioLegend	1:400
CD11b-BV421	101235	BioLegend	1:150 (flow)<br>1:250 (full-spectrum flow)

CD11c-BV510	117337	BioLegend	1:100
CD11c-PE	117307	BioLegend	1:100
CD11c-PE-CF594	562454	BD Biosciences	1:400
CD19-Biotin	115504	BioLegend	1:400
CD19-BUV737	612781	BD Biosciences	1:400
CD19-BV650	115541	BioLegend	1:250
CD25-PE	102007	BioLegend	1:200
CD45-APC	103111	BioLegend	1:200
CD45-BV605	563053	BD Biosciences	1:400
CD45-FITC	103107	BioLegend	1:200
CD45-PE	103106	BioLegend	1:250
CD45R (B220)-PE-Cy5	103210	BioLegend	1:800
CD49b (DX5)-PE-Cy5	15-5971-82	eBioscience	1:200
CD49R (B220)-PE-CF594	562290	BD Biosciences	1:800
CD68-FITC	137006	BioLegend	1:200
CD68-Al488	137012	BioLegend	1:250
CD88-PerCP-Cy5.5	135813	BioLegend	1:200
CD90.2-Biotin	105304	BioLegend	1:400
CD103-BV711	121435	BioLegend	1:400
CD115-PE	135505	BioLegend	1:100
CD161b/ c-BUV395	564144	BD Biosciences	1:400
CD183-PE	155903	BioLegend	1:200
CD192 (CCR2)-BV785	150621	BioLegend	1:200
CD194 (CCR4)- PE-Cy7	131213	BioLegend	1:200
CD206-PE-Cy7	141720	BioLegend	1:250
CD326-BUV737	741818	BD Biosciences	1:400
CX3CR1-BV711	149031	BioLegend	1:400
EPCAM-BV510	118231	BioLegend	1:200
F4/80-Al647	123122	BioLegend	1:400
F4/80-PERCP-Cy5.5	123127	BioLegend	1:100
F4/80-APC/ Fire 750	123152	BioLegend	1:250
LY-6C-Al647	128009	Biolegend	1:75 (IF)
LY-6C-APC	128015	BioLegend	1:200
LY-6C-APC-e780	47-5932-82	eBioscience	1:200
LY-6C-BV570	128030	Biolegend	1:500
LY-6G-BV510	127633	BioLegend	1:100
LY-6G-BV711	127643	BioLegend	1:250

LY-6G-V450	562366	BD Biosciences	1:200
MerTK-PE-Cy7	25-5751-82	eBioscience	1:100
MHC II-PE-Cy7	107629	BioLegend	1:200
MHC II-V500	562366	BD Biosciences	1:200
NK1.1-PE-Cy7	108713	BioLegend	1:200
Streptavidin-BUV496	612961	BD Biosciences	1:50
Siglec-F-FITC	155503	BioLegend	1:100
Siglec-F-PE/Dazzle 594	155529	BioLegend	1:250
TCRβ-PE	109207	BioLegend	1:100
Ter119-Biotin	116204	BioLegend	1:800
Tim-4-BV480	746499	BD Biosciences	1:250
XCR1-BV650	148220	BioLegend	1:400
ZEB1-Al647	40098	Cell Signaling	1:250
Zombie-NIR™ Dye	77184	BioLegend	1:750
