# Supplementary material for: Macrophages foster anti-tumor immunity by ZEB1-dependent cytotoxic T cell chemoattraction
Source: Commun Biol. 2025 Jul 1;8:976. doi: 10.1038/s42003-025-08339-7 (PMC12218307; doi:10.1038/s42003-025-08339-7)
Supplement: Supplementary file 3 — Description of Additional Supplementary Files [file 42003_2025_8339_MOESM3_ESM.pdf]

Description of Additional Supplementary Files

File name- Supplementary Data 1

File description- Enriched terms from all significant DEGs derived from RNA sequencing (FDR<0.05) of Zeb1-proficent (Ctrl) and ZEB1-deficient (Del) BMDMs in response to LPS or IL-4 and those term enrichments derived from DEGs shared between Ctrl and Del BMDMs.

File name- Supplementary Data 2

File description- Enriched terms from unique significant DEGs derived from RNA sequencing (FDR<0.05) of Zeb1-proficent (Ctrl) and ZEB1-deficient (Del) BMDMs in response to LPS or IL-4. Note that for the IL-4 stimulation, there was no significantly enriched terms except from genes downregulated exclusively in ZEB1-Del BMDMs.

File name- Supplementary Data 3

File description- The source data behind the graphs in the paper
