# Supplementary material for: Macrophages foster anti-tumor immunity by ZEB1-dependent cytotoxic T cell chemoattraction
Source: Commun Biol. 2025 Jul 1;8:976. doi: 10.1038/s42003-025-08339-7 (PMC12218307; doi:10.1038/s42003-025-08339-7)
Supplement: Supplementary file 7 — nr-reporting-summary [file 42003_2025_8339_MOESM7_ESM.pdf]

nature portfolio

Corresponding author(s): Harald Schuhwerk

Last updated by author(s): May 13, 2025

Reporting Summary

Nature Portfolio wishes to improve the reproducibility of the work that we publish. This form provides structure for consistency and transparency in reporting. For further information on Nature Portfolio policies, see our Editorial Policies and the Editorial Policy Checklist.

Statistics

For all statistical analyses, confirm that the following items are present in the figure legend, table legend, main text, or Methods section.

n/a	Confirmed	
☐	☒	The exact sample size (*n*) for each experimental group/condition, given as a discrete number and unit of measurement
☐	☒	A statement on whether measurements were taken from distinct samples or whether the same sample was measured repeatedly
☐	☒	The statistical test(s) used AND whether they are one- or two-sided<br>*Only common tests should be described solely by name; describe more complex techniques in the Methods section.*
☒	☐	A description of all covariates tested
☒	☐	A description of any assumptions or corrections, such as tests of normality and adjustment for multiple comparisons
☐	☒	A full description of the statistical parameters including central tendency (e.g. means) or other basic estimates (e.g. regression coefficient) AND variation (e.g. standard deviation) or associated estimates of uncertainty (e.g. confidence intervals)
☐	☒	For null hypothesis testing, the test statistic (e.g. *F*, *t*, *r*) with confidence intervals, effect sizes, degrees of freedom and *P* value noted<br>*Give P values as exact values whenever suitable.*
☒	☐	For Bayesian analysis, information on the choice of priors and Markov chain Monte Carlo settings
☒	☐	For hierarchical and complex designs, identification of the appropriate level for tests and full reporting of outcomes
☒	☐	Estimates of effect sizes (e.g. Cohen's *d*, Pearson's *r*), indicating how they were calculated

Our web collection on statistics for biologists contains articles on many of the points above.

Software and code

Policy information about availability of computer code

Data collection	For RNA isolation, cDNA synthesis and quantitative reverse transcriptase PCR, cells were washed with PBS and lysed with 350 µl RLT Plus from the RNeasy Plus Mini Kit (QIAGEN, 74136) before RNA isolation following manufacturer´s instructions. cDNA was synthesized using the RevertAid First Strand cDNA synthesis Kit (Thermo Fisher Scientific, K1622) according to manufacturer´s instructions. Bulk RNA sequencing of BMDM RNA (n=3 per condition) was performed by Novogene using poly(A) enrichment library preparation protocol and paired-end sequencing (PE150). The preprocessing of raw RNA-seq data (FASTQ files) was performed using the nf-core RNA-seq pipeline v.3.8.11. In particular, reads were adapter- and quality-trimmed using Trim Galore v.0.6.7 (https://github.com/FelixKrueger/TrimGalore). The reads were then mapped to the Ensembl mouse genome assembly GRCm39 (release 107) using STAR v.2.7.10a. For transcript-level read counting, Salmon v.1.5.2 was employed, relying on the Ensembl gene annotation file release 107. To generate gene-level counts, Salmon's transcript-level quantification files were processed using the R-package tximport v.1.22 within R v.4.0.3. Differential expression analysis for the RNA-seq data was performed using the DESeq2 package v.1.34.0. As DESeq2 design formula ~litter + group was utilized, where the group factor was created by merging together genotype (LysMCtrl/LysMΔZeb1) and treatment (LPS/IL-4). Fold change shrinkage was performed relying on the "ashr" method, made available within the DESeq2 package. GO term analysis was performed using Enrichr. For this, for all significantly differentially expressed genes (DEGs) from the indicated comparisons (adjusted p value: FDR<0.05) were used and the -log10 (FDR) values plotted with GraphPad Prism. The numbers of identified DEGs for the indicated comparisons that were used for GO term analyses are indicated in the Venn-like diagram in Fig. 5A, B.
Data analysis	- Bioluminescence signal was measured in the IVIS Spectrum In Vivo Imaging System (Perkin Elmer)<br>- CytoFlex Analyzer (Beckman Coulter). Data was analyzed using the CytExpert, Kaluza software (Beckman Coulter or FlowJo (BD).<br>- ImageJ (Fuji 1.53C), CellProfiler were used for quantification of histological, immunohistological and immunofluorescence images<br>- ChemiDocTM Imaging System (BioRad) was used for western blot and secretome data analysis<br>- For pan-cancer single cell sequencing analysis of myeloid cells, an online was utilized (http://panmyeloid.cancer-pku.cn). The dataset

'Pan_cancer scanorama_corrected' was selected and filtered for 'Macro', 'Mono', 'Monolike' and 'Myleoid' clusters. The Zeb1 feature plot (on UMAP clusters) was directly retrieved from the portal.
For murine pancreatic cancers, the FASTQ files of 76. Gabitova-Cornell et al., were aligned to the reference transcriptome CellRanger 7.1.0 and subjected to further processing using Seurat 5.0., involving quality control (number of genes detected in each cell > 300; total number of molecules detected per cell > 500; mitochondrial read count ratio cutoff <25%; Haemoglobin ratio <10%), normalization of counts using Seurat´s 'SCTransform', integration of the three datasets using Seurat´s 'RPCAIntegration', and clustering. Next, DECORDER annotation was used to annotate the individual cells, of which immune cells were UMAP sub-clustered. 'FindAllMarkers' (top 50 DEGs of a given cluster versus all other cell clusters) allowed annotation of individual immune cell types/clusters (B cells: CD2+;Bkl+;Fcmr+, T cells: Gata2+;Itk+; dendritic cells: Dcstamp+;Slamf9+, macrophages/granulocytes: Tlr4+;Ccrl2+). The feature plot in Fig. 1b was created using 'SCPubr' (https://enblacar.github.io/SCpubr-book/closing_remarks/Citation.html).
For CRC single cell sequencing analysis of myeloid cells, the built-in online tool from the Broad Institute´s 'single cell portal' (https://singlecell.broadinstitute.org/single_cell) was used to explore the dataset from. Plots and cell type annotations were directly retrieved from the portal. For myeloid subtype clustering, cell types were filtered for 'myeloid' cells (in Fig. 1c, Zeb1 feature plot), and, among those, for 'macrophage-like', 'monocytes' and 'granulocytes' (in Fig. 1d for comparison), using the respective available filters.
- LightCycler® 480 II Software 1.5.1 was used for qRT-PCR data analysis
- GraphPad Prism 9 was used for statistical analysis

For manuscripts utilizing custom algorithms or software that are central to the research but not yet described in published literature, software must be made available to editors and reviewers. We strongly encourage code deposition in a community repository (e.g. GitHub). See the Nature Portfolio guidelines for submitting code & software for further information.

Data

Policy information about availability of data

All manuscripts must include a data availability statement. This statement should provide the following information, where applicable:

- Accession codes, unique identifiers, or web links for publicly available datasets
- A description of any restrictions on data availability
- For clinical datasets or third party data, please ensure that the statement adheres to our policy

The RNA sequencing data is deposited at GEO under the accession GSE286348 and will be made available for public access upon publication of this study. Reviewer tokens have been provided.

Human research participants

Policy information about studies involving human research participants and Sex and Gender in Research.

Reporting on sex and gender	Not applicable
Population characteristics	Not applicable
Recruitment	Not applicable
Ethics oversight	Not applicable

Note that full information on the approval of the study protocol must also be provided in the manuscript.

Field-specific reporting

Please select the one below that is the best fit for your research. If you are not sure, read the appropriate sections before making your selection.

☒ Life sciences ☐ Behavioural & social sciences ☐ Ecological, evolutionary & environmental sciences

For a reference copy of the document with all sections, see nature.com/documents/nr-reporting-summary-flat.pdf

Life sciences study design

All studies must disclose on these points even when the disclosure is negative.

Sample size	For animal experiments power analysis was used to calculate the sample size a priori. For all experiments, number of biological replicates are indicated in figures and legends as well as as indivdual points within the graphs. Except for some flow cytometry measurements, sample size was n>=3.
Data exclusions	No data points were excluded
Replication	All experiments were done with biological replicates as indicated
Randomization	For animal studies, available mouse cohorts were grouped by geneotype and randomized for individual treatments

Blinding

The investigators were blinded for initial animal data collection on tumor/metastasis parameters. No blinding was applied for other experiments since investigators needed information about the groups to correctly perform and analyze the experiments.

Reporting for specific materials, systems and methods

We require information from authors about some types of materials, experimental systems and methods used in many studies. Here, indicate whether each material, system or method listed is relevant to your study. If you are not sure if a list item applies to your research, read the appropriate section before selecting a response.

Materials & experimental systems

n/a	Involved in the study
☐	☒ Antibodies
☐	☒ Eukaryotic cell lines
☒	☐ Palaeontology and archaeology
☐	☒ Animals and other organisms
☒	☐ Clinical data
☒	☐ Dual use research of concern

Methods

n/a	Involved in the study
☒	☐ ChIP-seq
☐	☒ Flow cytometry
☒	☐ MRI-based neuroimaging

Antibodies

Antibodies used

Primary antibodies:
Specificity, Host, Catalog no., Manufacturer, Dilution
α-Tubulin, mouse, T6199, Sigma-Aldrich, 1:5000
β-Actin mouse, A5441, Sigma-Aldrich, 1:5000
CD8, rabbit, 50389-T26, Sino Biologicals, 1:500
MCP1/CCL2, rabbit, PA115555, Thermo Fisher Sci., 1:600
CD68 (human), mouse, ab201973, Abcam, 1:100
CD68, rabbit, PA5-78996, Invitrogen, 1:600
cleaved Caspase-3, rabbit, 9664S, Cell Signaling, 1:200
F4/80, rat, MCA4976, Bio-Rad, 1:200
Ki67, rabbit, ab16667, abcam, 1:300
RAB6, rabbit, 9625T, Cell Signaling, 1:1000
RAB35, rabbit, 88244T, Cell Signaling; 1:1000
VAMP3, rabbit, 13640S, Cell Signaling, 1:1000
VAMP8 rabbit, 13060S, Cell Signaling, 1:1000
ZEB1, rabbit, HPA027524, Sigma-Aldrich, 1:1000 (IHC) / 1:2000 (WB)
ZEB1, rabbit, NBP1-05987, Novus Biologicals, 1:250
ZEB1, rabbit, E2G6Y, #70512, Cell Signaling, 1:400 (IF)
ZEB1, mouse, AMAb90510, Sigma-Aldrich, 1:300

Secondary antibodies:
AlexaFluor488 anti-rabbit IgG (H+L), goat, A11034, Sigma-Aldrich, 1:200
anti-rabbit-HRP polymer, goat, K4003, DAKO, 1:1
anti-rat-HRP, rabbit, A18915, life technologies, 1:500
CF640R anti-rabbit IgG (H+L), goat, SAB4600164, Sigma-Aldrich, 1:200
CF640R anti-mouse IgG (H+L), goat, SAB4600343, Sigma-Aldrich, 1:200
anti-rabbit-HRP (WB), goat, 111-035-144, Dianova, 1:10000
anti-mouse-HRP (WB), goat, 115-035-146, Dianova, 1:10000

FACS antibodies:
Specificity Catalog no. Manufacturer Dilution
TruStain FcX™ PLUS (anti-mouse CD16/32) 156603 BioLegend 1:200
Purified anti-mouse CD16.2 (9E9) 149502 BioLegend 1:400
InVivoMAb anti-mouse CD16/CD32 (2.4G2) BE0307 Bio X Cell 1:400
B220-APC 103211 BioLegend 1:200
CCR3-APC 144511 BioLegend 1:200
CD3e-Biotin 100304 BioLegend 1:200
CD3e-Al647 100322 BioLegend 1:200
CD4-Al488 100532 BioLegend 1:50
CD4-APC-Fire750 100568 BioLegend 1:400
CD4-BV605 100547 BioLegend 1:200
CD8a-Al647 100727 BioLegend 1:200
CD8a-BV570 100740 BioLegend 1:400
CD11b-Al700 101222 BioLegend 1:400
CD11b-BV421 101235 BioLegend 1:150 (flow)
1:250 (full-spectrum flow)
CD11c-BV510 117337 BioLegend 1:100
CD11c-PE 117307 BioLegend 1:100

CD11c-PE-CF594 562454 BD Biosciences 1:400
CD19-Biotin 115504 BioLegend 1:400
CD19-BUV737 612781 BD Biosciences 1:400
CD19-BV650 115541 BioLegend 1:250
CD25-PE 102007 BioLegend 1:200
CD45-APC 103111 BioLegend 1:200
CD45-BV605 563053 BD Biosciences 1:400
CD45-FITC 103107 BioLegend 1:200
CD45-PE 103106 BioLegend 1:250
CD45R (B220)-PE-Cy5 103210 BioLegend 1:800
CD49b (DX5)-PE-Cy5 15-5971-82 eBioscience 1:200
CD49R (B220)-PE-CF594 562290 BD Biosciences 1:800
CD68-FITC 137006 BioLegend 1:200
CD68-Al488 137012 BioLegend 1:250
CD88-PerCP-Cy5.5 135813 BioLegend 1:200
CD90.2-Biotin 105304 BioLegend 1:400
CD103-BV711 121435 BioLegend 1:400
CD115-PE 135505 BioLegend 1:100
CD161b/ c-BUV395 564144 BD Biosciences 1:400
CD183-PE 155903 BioLegend 1:200
CD192 (CCR2)-BV785 150621 BioLegend 1:200
CD194 (CCR4)- PE-Cy7 131213 BioLegend 1:200
CD206-PE-Cy7 141720 BioLegend 1:250
CD326-BUV737 741818 BD Biosciences 1:400
CX3CR1-BV711 149031 BioLegend 1:400
EPCAM-BV510 118231 BioLegend 1:200
F4/80-Al647 123122 BioLegend 1:400
F4/80-PERCP-Cy5.5 123127 BioLegend 1:100
F4/80-APC/ Fire 750 123152 BioLegend 1:250
LY-6C-Al647 128009 Biolegend 1:75 (IF)
LY-6C-APC 128015 BioLegend 1:200
LY-6C-APC-e780 47-5932-82 eBioscience 1:200
LY-6C-BV570 128030 Biolegend 1:500
LY-6G-BV510 127633 BioLegend 1:100
LY-6G-BV711 127643 BioLegend 1:250
LY-6G-V450 562366 BD Biosciences 1:200
MerTK-PE-Cy7 25-5751-82 eBioscience 1:100
MHC II-PE-Cy7 107629 BioLegend 1:200
MHC II-V500 562366 BD Biosciences 1:200
NK1.1-PE-Cy7 108713 BioLegend 1:200
Streptavidin-BUV496 612961 BD Biosciences 1:50
Siglec-F-FITC 155503 BioLegend 1:100
Siglec-F-PE/Dazzle 594 155529 BioLegend 1:250
TCRβ-PE 109207 BioLegend 1:100
Ter119-Biotin 116204 BioLegend 1:800
Tim-4-BV480 746499 BD Biosciences 1:250
XCR1-BV650 148220 BioLegend 1:400
ZEB1-Al647 40098 Cell Signaling 1:250

Validation

Antibodies were validated by knockout cell lines, manufacturer's web page including datasheets or listed publications, own previous studies and/or experience, subcellular localization of staining

Eukaryotic cell lines

Policy information about cell lines and Sex and Gender in Research

Cell line source(s)

Cell line derivatives were generated in this study from mouse colon and cell lines CMT93 and MC38 as well as from a pancreatic cancer cell line (KPC) generated and published by us previously (Krebs et al., Nature Cell Biology, 2017)

Authentication

cells have not been authenticated

Mycoplasma contamination

Cells were routinely tested for mycoplasma contamination by MycoAlert Mycoplasma Detection Kit (LT07-218; Lonza) and no contamination was observed throughout the study

Commonly misidentified lines
(See ICLAC register)

not applicable

Animals and other research organisms

Policy information about studies involving animals; ARRIVE guidelines recommended for reporting animal research, and Sex and Gender in Research

Laboratory animals

Animals in this study were generated in the facilities of FAU, Erlangen and kept on a C57BL/6 background (with the exception of

Laboratory animals	NOD.Cg-Prkdcscid Il2rgtm1Wjl/SzJ (Nod-Scid-gamma, NSG))
Wild animals	Not applicable
Reporting on sex	This study did not focus on sex differences. Cohorts for experiments were chosen by genotype, availability and age, independent of the sex. Nevertheless, roughly same numbers of males and females were used for the experiments.
Field-collected samples	No samples were collected from the field.
Ethics oversight	Animal husbandry and all experiments were performed according to the European Animal Welfare laws and guidelines. The protocols were approved by the local committee on ethics of animal experiments.

Note that full information on the approval of the study protocol must also be provided in the manuscript.

Flow Cytometry

Plots

Confirm that:

☒ The axis labels state the marker and fluorochrome used (e.g. CD4-FITC).

☒ The axis scales are clearly visible. Include numbers along axes only for bottom left plot of group (a 'group' is an analysis of identical markers).

☒ All plots are contour plots with outliers or pseudocolor plots.

☒ A numerical value for number of cells or percentage (with statistics) is provided.

Methodology

Sample preparation	After opening the abdomen and thorax, about 300 µl blood was slowly extracted from the right ventricle of the heart with an EDTA flushed syringe and added to 14 ml Hank´s solution. Samples were centrifuged for 10 min at 1400 rpm and all but 3 ml supernatant was removed. After blood harvesting, mice were perfused with 0.9% NaCl and organs collected in ice-cold PBS. Organs were minced with scissors and incubated in 10 ml digestion mix (DMEM/ F-12 containing 0.05% Collagenase D (Sigma-Aldrich, 11088858001), 0.3% Dispase II (Sigma-Aldrich, D4693) and 0.05% DNase I (Sigma-Aldrich, 10104159001) for 25 mins at 37 °C with gentle agitation. Digestion was stopped by adding 30 ml ice-cold PBS, the samples were filtered through a 70 µm cell strainer and centrifuged at 1200 rpm at 4 °C for 5 min. The cell pellet was resuspended in 3 ml ACK Lysis buffer. After 3 min, erythrocyte lysis was stopped with 27 ml PBS and the samples centrifuged for 5 min at 1200 rpm at 4 °C. 1x106 cells were blocked in 50µl TruStain FcX PLUS in PBS for 15 min a RT. 50 µl of 2x FACS antibody mix was added and incubated for 20 min at 4 °C in the dark. Details about all antibodies are provided in table S3. Following centrifugation for 5 min at 1200 rpm at 4 °C, cell pellets were resuspended in 500 µl FACS buffer. Samples were analyzed in the CytoFlex Analyzer (Beckman Coulter). Data was analyzed using the CytExpert or Kaluza software (Beckman Coulter).<br>For flow cytometry cross-tissue UMAP clustering, samples were stained in 50µL of FACS buffer (PBS+2% FCS) containing purified FC blocking antibodies (clone 2.4G2 and clone 9E9) as well as the monoclonal biotin coupled antibodies for a total of 20 minutes at 4°C. Next, samples were washed three times with FACS buffer. Subsequently, cells were stained with monoclonal fluorescently-labeled antibodies and streptavidin-BUV496. Details about all antibodies are provided in table S3. After three washing steps, samples were supplemented with FACS buffer containing DAPI in a final concentration of 50 ng/mL and analyzed using a LSR Fortessa SORP (BD). Data was analyzed using FlowJo (BD). 5000 (myeloid panel) or 2150 (lymphoid panel) cells per tissue were randomly selected from the pool of CD45+ cells, combined to form an analysis sample and UMAP was performed. The individual samples were again separated by tissue and the populations identified by gating.<br>Surface staining of CD192, CD194 and CD183 on splenic CD8+ T cells (enriched via magnetic bead separation as described below) was performed as described above but for 30 min at 37°C. For details on antibodies see table S3. Samples were analyzed by full spectrum flow cytometry using a Northern Lights™ cytometer (Cytek Biosciences). Following spectral unmixing using the built-in acquisition software (Cytek Bioscience), the data was analyzed using FlowJo (BD). For flow cytometry of intracellular ZEB1, organs were collected as described above and isolated using the Tumor Dissociation Kit (130-096-730, Miltenyi Biotec) according to the manufacturer's instructions. Following live/dead discrimination using Zombie NIR (423105, Biolegend), surface and intracellular staining after (Fc block) was performed using the FoxP3 Staining Buffer Set (130-093-142, Miltenyi Biotec) according to the manufacturer's instructions, using the antibodies listed in table S3. Data was acquired and analyzed by full spectrum flow cytometry as described above.
Instrument	Samples were analyzed in the CytoFlex Analyzer (Beckman Coulter). Data was analyzed using the CytExpert, Kaluza software (Beckman Coulter) or Flojo (BD).<br>For flow cytometry cross-tissue UMAP clustering, samples were analyzed using a LSRFortessa SORP (BD). Data was analyzed using FlowJo (BD).<br>The samples from surface staining of CD192, CD194 and CD183 on splenic CD8+ T cells (enriched via magnetic bead separation as described below), and from flow cytometry of intracellular ZEB1 were analyzed by full spectrum flow cytometry using a Northern Lights™ cytometer (Cytek Biosciences). Following spectral unmixing using the built-in acquisition software (Cytek Bioscience), the data was analyzed using FlowJo (BD).
Software	Data was analyzed using the CytExpert, Kaluza software (Beckman Coulter) or Flojo (BD).
Cell population abundance	Sorted cells were reanalyzed by same gating strategy and purity were >90%.

Gating strategy	Depending on the experiment. Gating strategies are depicted, explained and indicated in the respective figures and corresponding legends.

☒ Tick this box to confirm that a figure exemplifying the gating strategy is provided in the Supplementary Information.
